# Comparing the Therapy of Otomycosis Using Clotrimazole with Iodine Tincture: A Clinical Trial 

**DOI:** 10.22038/ijorl.2021.51647.2751

**Published:** 2021-07

**Authors:** Mohammad Reza Mofatteh, Mahboubeh Ahi Fersheh, Fatemeh Nikoomanesh, Mohammad Hasan Namaei

**Affiliations:** 1 *Department of Ears Nose and Throat, School of Medicine, Birjand University of Medical Sciences, Birjand, Iran.*; 2 *Student Research Committee, Birjand University of Medical Sciences, Birjand, Iran.*; 3 *Infectious Diseases Research Center, Birjand University of Medical Sciences, Birjand, Iran.*

**Keywords:** Antifungal agents, Clotrimazole, Iodine tincture, Otomycosis, Mycosis

## Abstract

**Introduction:**

Otomycosis, as a common superficial fungal infection, is the term to infection of external auditory canal. Despite numerous studies on diverse antifungal agents, there is no common consent on effective agent for treatment of otomycosis. Therefore, the purpose of this study is compared therapy of otomycosis using two therapeutic agents; clotrimazole and iodine tincture.

**Materials and Methods:**

This research is a clinical trial study included 160 patients who were presented otomycosis. All patients were randomly assigned into two therapeutic groups of clotrimazole and Iodine Tincture (80 cases in each group). The results of response to thrapy were evaluated on 4, 10, and 20 days. Statistical analyses were performed using Independent-Samples t-test, Chi-Square, and Fishers҆ Exact tests in SPSS software v.18, in 0.05 significant level.

**Results:**

Fungal species were isolated including *Aspergillus* (72.5%) and *Candida albicans* (22.5%). After 4^th^day of treatment, 7.5% of the tincture group and 11.2% of the clotrimazole group revealed a good response to treatment (*P=0.30*). A good response to treatment was observed in35.0 and 41.2% of the patients on 10^th^ day of treatment (*P=0.44*); and in 67.5 and 62.5% of the patients on 20^th^ day of treatment (*P= 0.20*). There was no significant relationship between the two therapeutic arms.

**Conclusion:**

In this study, both clotrimazole and tincture showed the identical therapeutic efficacy on otomycosis. Our findings suggested that tincture can be used as a supplementary antifungal option for treatment of otomycosis.

## Introduction

Otomycosis, as a common superficial fungal infection, is the term to infection of external auditory canal. Prevalence of the etiologic agent depends on the geographical area (as hot and moist climates) and predisposing factors such as temperature and humidity (1). Otomycosis has been reported in 30–90% of otitis external cases. It is a global disease, but is more common in adults than in kids and in females than in males (2, 3). A wide range of fungi cause otomycosis. The major etiologic agent for otomycosis is *Aspergillus* and *Candida* genera. Beside, the third group is included *Dermatophytes* which are caused otomycosis. In rare cases, other saprophytic fungi including *Fusarium* spp., *Penicillium* spp. *Mucor *spp., and *Geotrichum *spp. have been isolated from otitis discharge (4,5). 

Otomycosis is generally a common superficial infection, that can be associated with several risk factors include traumatic inoculation by sharp objects, presence of cerumen, dry and dusty environment, humidity and moisture, swimming, genetic factors, and ear surgery (6,7).Today otomycosis treatment has made challenges both for patients and physicians despite the need for the long process of treatment and possibility of relapse (8). Treatment recommendations involves consistent debridement of fungal agents from the ear canal, combined with application of local and systemic antifungal (9). 

The anti-fungals used currently for otomycosis therapeutic include clotrimazole, bifonazole, miconazole, and tolnaftate, though the exact proper treatment has still remained unknown (10-13). Anti-fungals such as clotrimazole or nystatin can be efficient against *Candida*, but it does not cover *Aspergillus* (14). Some researchers believe that the selection of treatment for otomycosis relate to the discernment of fungal species and its antifungal susceptibility (9). 

Due to the increased prevalence of drug resistance among *Aspergillus* spp. as an agent of otomycosis, the appropriate treatment regimen should be selected. Furthermore, extensive and dispensable use of antifungal causes excessive growth of the organism in the external canal ear.

Many *in-vivo* and *in-vitro* studies have been conducted on various antifungal agents as an alternative to conventional therapeutics (15-17). Various agents have been as well as used clinically with uncertain rate of effectiveness. However, no study has revealed the affect of iodine tincture as an antifungal agent in treatment of otomycosis. It is introduced that iodine is normally used as an antiseptic agent in surgical wards, and no resistance has been documented for this agent so far. In previous study conducted by Mofatteh et al. (2017) in a study compared recovery rate of otomycosis by betadine (povidone-iodine) with clotrimazole (18). Povidone-iodine (betadine) is a chemical complex consisting of povidone, hydrogen, and elemental iodine (17). Iodine tincture is an antiseptic agent. It is usually composed of 2–7% of elemental iodine, along with potassium iodide or sodium iodide, dissolved in a mixture of ethanol and water (19-21). 

Tincture solutions are characterized by the presence of alcohol. Iodine tincture is one of the most widely used skin disinfectants applied before surgery and no cases of toxicity have been reported so far (21).

Accordingly, this research was done to compare improvement rate of otomycosis treated with topical medications; clotrimazole and iodine tincture. Relative frequency of response to treatment in both groups was evaluated during three periods. Finally, it was tested whether relative frequency has a significant difference regarding response to treatment when comparing clotrimazole with iodine tincture based on clinical observations regarding effectiveness of each agent.

## Materials and Methods

**Study participants**

This research is a clinical trial conducted on 172 patients whom were definitely diagnosed otomycosis. This research has been approved by the Ethics Committee of the Birjand University of Medical Sciences and registering the study in the Iranian registry of clinical trials (IRCT) with registration number of IRCTID: IRCT2016073020484N4.

All study subjects were selected from patients referred to the ear, nose, and throat (ENT) Clinic of the Imam Reza Hospital affiliated to the Birjand University of Medical Sciences (Birjand, South Khorasan Province, Iran) during the first 6months in 2016.Perforation of the tympanic membrane (TM) was prior to recruitment. The inclusion criteria for this study were having complaints such as pain, itching, inflammatory puritis, deafness, and persistent discharge. Patients who were having precedent of therapy with antibiotics and ear surgery, otitis media with restriction of external ear canal, chronic mucus from ear, and who had corticosteroids, and external ear anomaly were excluded in this study. The targets of the research were clarified to the subjects and informed consents were signed before participating in the study. 

After obtaining the informed written consent, demographic characteristics, including: age, gender, etc., were recorded. A total of 172 patients were taken part into the research, and they were randomly allocated into one of the two treatment groups (86cases in each group).

**Isolates and identification**

Samples were removed using special speculum from ear canal. Then, to diagnose infection, all samples was placed in a sterile container, transport to the mycology laboratory.

**Mycological diagnosis**

Portions of samples were prepared using KOH method for direct examination, and the observation of fungal elements was diagnosed otomycosis by microscopic examination. Other portions of the samples as previously described(18), were cultured on two plates of Sabouraud Dextrose Agar (SDA, Merck, Germany) included chloramphenicol (Fina Daru, Iran) and incubated at 25°C and 37°C for two weeks. Yeasts were also detected with germ tubes production in serum and the chlamydospore formation on corn meal agar (Merck, Germany& Sigma-Aldrich, Germany) supplemented with tween 80.

**Monitoring of recovery rate**

In this study, all the patients underwent external auditory canal (EAC) cleaning and debridement. A patient group was treated with Iodine tincture according to the treatment protocol previously described in the literature (18),For this purpose, iodine tincture was prepared from mixing 2% iodine solution with 100ccof 47 %alcohol and2.4 g ofiodine potassium. The patient’s external canal ear was washed by the physician using 10 ml of iodine tincture with a syringe at each visit. Participates in clotrimazole group took 8 drops of 1% clotrimazole (ear drop) each 8 h after cleaning the ear canal with suction. The amount of response to therapy was evaluated on four, 10, and 20 days by an ENT specialist. The response rate to treatment was graded into three groups associated with the clinical presentation: good response (dry ear canal and TM and decrease of discharge), partial response (slightdischarge but not dry), and no response (hypersecretion in the EAC). If certain recovery was not taken, the treatment process was discontinued, and otherwise therapy continued. Eventually, non- treatment was considered as resistance cases after20th day, and therapy regimen was done by tolnaftate and violet de gentian.

**Statistical analysis**

Data was statistically analyzed by used SPSS (V.18) software. Descriptive statistics, Chi-square test and t-test were used for analysis of distribution frequency and for the inferential part. In all of statistical analyses P-value<0.050 was considered significant.

## Results

In present study, 12 out of 172 subjects were excluded from the research because of side effects, such as burning and itching. Therefore, 160 patients with diagnosed otomycosis were considered in two groups of clotrimazole and iodine tincture (80 cases per each group).Random allocation was made to match the two study groups in terms of age and gender. Response to treatment were characterized into three groups according to clinical presentation: (i) good response (dry EAC and TM and no secretion), (ii) partial response (lack discharge but not dry), and (iii) no response (hypersecretion in the EAC).Consequently, the patients who did not respond appropriately to tincture treatment, standard treatment were used. Mean age of the subjects in two treatment groups (clotrimazole and Iodine tincture) was equal to 32.56±13.98 and 34.15±14.60 years old, respectively (Table.1). 

In both groups, women accounted for the majority of participants. In this study, *Aspergillus* was identified in 116out of 160 patients (72.5 per cent) isolates and *Candida albicans* was detected in 44 patients҆ isolates. Co-infection or double infection was not detected (Table.1).

**Table1:** Demographic characteristics and frequency of fungi isolated patients with otomycosis

**Demographic characteristics**	**Clotrimazole group (%)**	**Iodine tincture (%)**	**P-value**
Age ^*^ Gender Male Female Fungal agent *Aspergillus*spp *Candida albicans*	34.15±14.6 35(43.7) 45(56.3) 58(72.5) 22(27.5)	36.52±13.98 29(36.3) 51(63.7) 58(72.5) 22(27.5)	0.29 0.33 1.0

^*^Values in table are mean ± SD

Overall, the results revealed that there was no significant difference between age (*P*=0.29), gender (*P*= 0.33) and causative agent of otomycosis (*P*=1.0) of the two groups. Evaluation of recovery rate after four days in tincture group showed that 7.5% of the patients detected good response and in clotrimazole group, 11.2% of the patients showed a good response (dry ear canal and TM and no discharge) to treatment. As well, parcial response (lack discharge but not dry) to treatment was obtained as 51.2 and 58.7% for tincture and clotrimazole groups, respectively(*P*= 0.30). Evaluation of recovery rate after 10days showed a good response by 35 and 41.2%for tincture and clotrimazole groups, respectively (*P*= 0.44).

Finally, on 20^th^day of treatment, good response was shown in the treatment groups with clotrimazole and tincture by 62.5 and 67.5%, respectively (*P*= 0.20).The reports regarding response to treatment each of groups are shown in Table 2. There was no significant relationship difference between two treatment groups of clotrimazole and Iodine tincture on 4^th^, 10^th^, and 20^th^ day (*P*<0.05).

**Table 2:** Comparison of response to treatment after define periods

**Course of treatment**	**Type of treatment**	**Response to treatment** ^*^			***P-value***
		good response%	Partial response%	No response%	
4days	Tincture clotrimazole	6(7.5) 9(11.2)	41(51.2) 47(58.7)	33(41.2) 24(30.0)	0.30
10days	Tincture clotrimazole	28(35.0) 33(41.2)	43(53.7) 35(43.7)	9(11.2) 12(15.0)	0.44
20days	Tincture clotrimazole	54(67.5) 50(62.5)	23(28.7) 21(26.2)	3(3.7) 9(11.2)	0.20

^*^(i) good response, (ii) partial response and (iii) no answer

Rate of recurrence after 10days of treatment with tincture was measured as 6.25% and in clotrimazole group; it was equal to 7.5%. Not observed significant difference in terms of rate of recurrence between two study groups (*P=*0.75). Also, recurrence rate was equal to 8.75 and 7.5% in tincture and clotrimazole groups, respectively after 20 days, showing no significant difference (*P*= 0.77, Table.3).

**Table3:** Comparison of relapse rates 10 and 20 days after treatment in patients by type of treatment

**treatment**	**Recurrence 10 days after treatment**		**Recurrence 20 days after treatment**		**Total(%)**
	Positive (%)	Negative (%)	Positive (%)	Negative (%)	
Clotrimazole	6(7.5)	74(92.5)	6(7.5)	74(92.5)	80(100)
Tincture	5(6.25)	75(93.5)	7(8.75)	73(91.25)	80(100)
Total	11(6.88)	149(93.12)	13(8.13)	147(91.87)	160(100)

## Discussion

***Fungal infection of the otitis, otomycosis, is a term of external auditory canal fungal infection which frequently reported by ENT specialists and it often diagnosis by clinical presentation.*** **Aspergillus** ***and*** **Candida** ***are the prevalent fungi that identified pathogenic saprophytes involved in otomycosis (*** 1 ***,*** 22 ***). In the present study, the most etiological identified agents were*** **Aspergillus** ***spp 72.5% (n=116/160) and*** **Candida albicans** ***27.5% (n=44/160) (*** Table.3 ***). In the study conducted by Kour et al. they reported,*** **Aspergillus** ***41.1% (*** **A. niger** ***36.9%) and*** *C.* ***albicans 8.2% were isolated from 72 patients (*** 23 ***).***

In another study conducted by Kenneth et al (2002), they reported that *Aspergillus* and *C. albicans* were isolated from 75% and 70% of twenty patients suffering external ear fungal infection (24)*. *Pradham et al. (2003) and Kiakujori et al. (2014) reported that commonly fungal species which were frequent isolated, are* Aspergillus* and *Candida* (22,25).The results of our research were consistent with the others research which mention before. *Aspergillus *spp are one of the opportunistic fungal which are commonagent of invasive fungal infections, particularly otomycosis. Therefore, *Apergillus* spp is known as the great majority of mycelia fungi cases of otitis external. Hence, selection of an appropriate treatment regimen against any given fungal species with high levels of resistance to therapy and relapse is very important. There is limited clinical evidence regarding specific antifungal treatment and also there is no information on duration of treatment. Several studies have been conducted in relation to the effect of antifungal therapy. Studies done in this area have introduced azole as the most effective antifungal agent, followed by tolnaftate and nystatin in treatment otomycosis (5).In general, clotrimazole, ketoconazole, itraconazole, terbinafine, and miconazole are applied as azoles for treatment of otomycosis (12). Among them, clotrimazole is known to be the most widely antifungal for treatment of otomycosis by acting on a wide range of yeast and filamentous species. Recently, an outbreak of fungal species has been reported that are resistant to clotrimazole, which has led to recurrences of the disease. Even If completely treated, a significant percentage of the patients experience a recurrence of the infection (12). For finding an effective agent in treatment of otomycosis, therapeutic aspects, such as length of treatment, ease of treatment protocol, and economic issues should also be considered (6).

Although, many *in vitro* studies were demonstrated affect of numerous antifungal agents, there is no common consent on effective therapeutic for otomycosis. Various agents for example, acetic acid, isopropylalchol (26), salicylic acid (27), tolnaftate (28), and boric acid have been also applied clinically with variable rate of success(6).

In the present study, iodine tincture was selected as it is inexpensive and easily available, and also it is chemically stable, and bacteria and fungi resistance to this agent is not reported yet (29). Iodine tincture 2% well known as broad-spectrum antiseptic and whit high-performance, which leading to oxidation of genes of cytoplasmic proteins and denaturation of amino acids, in the bacteria. Iodine tincture is in despite of active against Gram-positive bacteria, it has inhibitory effects on fungi (30). Due to its ethanol content, iodine tincture has been reported to display very high antimicrobial activity against microorganisms including *Aspergillus* spores (29). Usually, only a few human cases of iodine poisoning have been reported, in whom topical inflammation has been found as adverse effects, and toxic dose of iodine for humans was approximately to be evaluated between 2-4g (20). Although it is a highly effective antiseptic, its use is limited because of its irritant properties such as, allergic response (Local burning and itching) (21).

After the curative period, recovery rate was monitored in three courses on 4, 10 and 20 days. The results on the fourth days after treatment with tincture and clotrimazole had partial responses of 51.2% and 58.7% respectively. On the tenth day, 53.7% of patient showed partial response to tincture and 41.2% had a good response to clotrimazole. Eventually, on the 20^th^ day, both arms of tincture and clotrimazole revealed good responses of 67.5% and 62.5% respectively. Comparison of the results of the two treatment regimns showed no significantly relationship between treatment of clotrimazole and tincture (Table.2). However, rarely, a study has been done to evaluate the function of iodine compounds as an antifungal in treatment of otomycosis. There have been just two studies about effectiveness of povidone-iodine in therapy of otomycosis. In the study, conducted by Philip et al. (2013), effectiveness of povidone-iodine 7.5% was evaluated in treatment of otomycosis compared to clotrimazole 1% solution used topically (17). Their results demonstrated improvements in both therapeutic arms and they suggested that povidone-iodine can be used effective antifungal agent in treatment of otomycosis. Mofatteh et al., (2017) reported that efficacy of betadine (povidone-iodine) 10%was the same compared to clotrimazole 1% ear drop used as a topical medication, for treatment of otomycosis (18).

Traditionally, iodine compounds have been used for skin disinfection, and to date, there are no document that showed development of resistance to these agents, which is reason of concern in this cases (31,32). Effective treatment of otomycosis, recommended to application of the appropriate antifungal agents, besides elimination fungal debris from the ear canal and omission of predisposing factors. Rapid and timely diagnosis of the disease and considering the possibility of resistance to treatment for chronic infections are important. 

In this study, clotrimazole and iodine tincture were applied as topical two therapeutic regimens for treatment of otomycosis. Clotrimazole is a member of azole group which widely used to treatment of mycosis. Iodine tincture is also known antiseptic that is easily available and effectiveness has been proven on infection of otitis external. Therefore, iodine tincture can be used as a supplementary antifungal option for treatment of otomycosis**, **especially in developing countries, inasmuch as, its economical, and the lowest level of cytotoxicity (20,21).

The present study also had some limitations, response to treatment was categorized based on clinical evidence and after the treatment period and culture of debris was not performed. As well as, detection of fungal agents isolated from patients' samples was performed by morphological and phenotypic diagnostic methods. Therefore, recommended that rapid molecular methods can be used to identify the species of fungal isolates. In addition, the minimum inhibitory concentration determined iodine tincture for optimal treatment.

## Conclusion

According to the results, iodine tincture is known as an antifungal agent, which is accessible and affordable, whose effect has been proven on treatment of otomycosis. Indiscriminate use of the antibiotics and antifungal leads to resistance to them, except for tincture as no bacterial and fungal resistance has been reported to it. Our findings suggested that tincture can be applied as a supplementary antifungal option for treatment of otomycosis.
